# Mental Health of Young Physicians in China During the Novel Coronavirus Disease 2019 Outbreak

**DOI:** 10.1001/jamanetworkopen.2020.10705

**Published:** 2020-06-01

**Authors:** Weidong Li, Elena Frank, Zhuo Zhao, Lihong Chen, Zhen Wang, Margit Burmeister, Srijan Sen

**Affiliations:** 1 Brain Science and Technology Research Center, Key Laboratory for the Genetics of Development and Neuropsychiatric Disorders (Ministry of Education), Shanghai Key Laboratory of Psychotic Disorders, Institute of Psychology and Behavioral Science, Bio-X Institutes, Shanghai Jiao Tong University, Shanghai, China; 2 Michigan Neuroscience Institute, University of Michigan, Ann Arbor; 3 Department of Hospital Administration, Shanghai Jiao Tong University School of Medicine, Shanghai, China; 4 Shanghai Mental Health Center, Shanghai Jiao Tong University School of Medicine, Shanghai, China; 5 Department of Psychiatry, Department of Computational Medicine and Bioinformatics, University of Michigan, Ann Arbor

## Abstract

This cohort study assesses anxiety, depression, mood, and fear of workplace violence in a cohort of young physicians in China before and during the coronavirus disease 2019 outbreak.

## Introduction

With more than 3 million cases worldwide, the novel coronavirus disease 2019 (COVID-19) poses a growing global public health challenge.^1^ Medical personnel disproportionately bear the additional physical and psychological burdens associated with pandemics, yet the mental health implications of COVID-19 for physicians are unknown.^2,3^ In this cohort study, we assessed anxiety, depression, mood, and other established factors associated with mental health problems in a cohort of young physicians in China before and during the outbreak.

## Methods

 The ethics committees of Shanghai Jiao Tong University and the University of Michigan approved this study. All participants provided written informed consent and were compensated ¥25 (as of May 7, 2020, ¥1 = $0.14 US). This study follows the American Association for Public Opinion Research (AAPOR) reporting guideline.

Training physicians from 12 Shanghai hospitals who enrolled in the prospective Intern Health Study in August 2019 completed surveys 2 weeks before beginning residency and again at 3 months (before the COVID-19 outbreak) and 6 months (during the COVID-19 outbreak) that assessed anxiety (Generalized Anxiety Disorder–7 scale), depression (Patient Health Questionnaire–9), and workplace violence.^4,5^ Mood valence (rated from 1 to 10, with higher scores indicating better mood) was measured daily via a mobile smartphone application. The same protocol was used to collect data in the prior 2018 to 2019 residency cohort.

A series of random effect mixed models were fitted to assess changes in Generalized Anxiety Disorder–7, Patient Health Questionnaire–9, and mood scores and experience, observation, and fear of workplace violence between quarter 1 and quarter 2 for both the 2018 to 2019 and 2019 to 2020 cohorts. Preresidency baseline (mood score, Generalized Anxiety Disorder–7 and Patient Health Questionnaire–9 total scores, and a personal history of depression) and within-residency (work hours, sleep duration, and experience, observation, and fear of violence before and after the outbreak) factors that have previously been associated with depression and anxiety in training physicians were included in the models.^6^ All analyses were performed using SAS statistical software version 9.4 (SAS Institute). Statistical significance was calculated with generalized linear mixed models. A 2-tailed *P* < .05 was considered statistically significant. Data analyses were performed in March and April 2020.

## Results

Of the 1037 invited residents, 726 (70%) agreed to participate in the study. Of those 726 residents, 385 (53%) completed the quarter 1 or quarter 2 surveys and were included in the analysis (247 women [64%]; median age, 25 years [interquartile range, 23-28 years]). For the 2019 to 2020 cohort, daily mood scores decreased statistically significantly between quarter 1 and quarter 2 (β = −0.50; 95% CI, −0.80 to −0.20; *P* = .002). In parallel, scores for symptoms of depression (β = 0.61; 95% CI, 0.08 to 1.14; *P* = .02) and anxiety (β = 0.64; 95% CI, 0.17 to 1.12; *P* = .008) increased statistically significantly during this period (Table). Furthermore, fear of violence (odds ratio, 2.36; 95% CI, 1.56 to 3.57; *P* < .001) and observation of violence from patients or their families (odds ratio, 3.63; 95% CI, 2.50 to 5.27, *P* < .001) increased statistically significantly (Table). In contrast, there were no statistically significant changes in mood, anxiety, or depressive symptoms or workplace violence status between quarter 1 and quarter 2 for the 2018 to 2019 cohort (Figure).

**Table.** Differences in Mood Score, Symptoms of Anxiety and Depression, and Workplace Violence Between Quarter 1 and Quarter 2 by Cohort

Cohort	Q1	Q2	β or OR (95% CI)^a^	*P* value	Q1	Q2	β or OR (95% CI)^a^	*P* value	Q1	Q2	β or OR (95% CI)^a^	*P* value
	Mood				GAD-7				PHQ-9			
2018-2019^b^	6.70	7.25	0.24 (−0.43 to 0.91)	.43	5.30	5.43	0.01 (−0.57 to 0.58)	.98	6.25	6.19	−0.32 (−0.93 to 0.29)	.30
2019-2020^b^	7.07	6.80	−0.50 (−0.80 to −0.20)	.002	4.33	5.43	0.64 (0.17 to 1.12)	.008	5.17	5.77	0.61 (0.08 to 1.14)	.02
	Fear of violence				Observed violence				Experienced violence			
2018-2019^c^	21.27	14.68	0.63 (0.38-1.03)	.06	25.08	24.77	0.92 (0.60-1.40)	.68	6.35	8.72	1.46 (0.75-2.86)	.27
2019-202^c^	17.76	30.31	2.36 (1.56-3.57)	<.001	22.12	49.48	3.63 (2.50-5.27)	<.001	7.48	8.01	1.13 (0.60-2.15)	.70

Abbreviations: GAD-7, Generalized Anxiety Disorder–7; OR, odds ratio; PHQ-9, Patient Health Questionnaire–9; Q1, quarter 1; Q2, quarter 2.

^a^ β values refer to the change between quarters 1 and 2 for all variables.

^b^ Data are mean scores.

^c^ Data are percentage of participants who answered yes on the survey.

**Figure.**
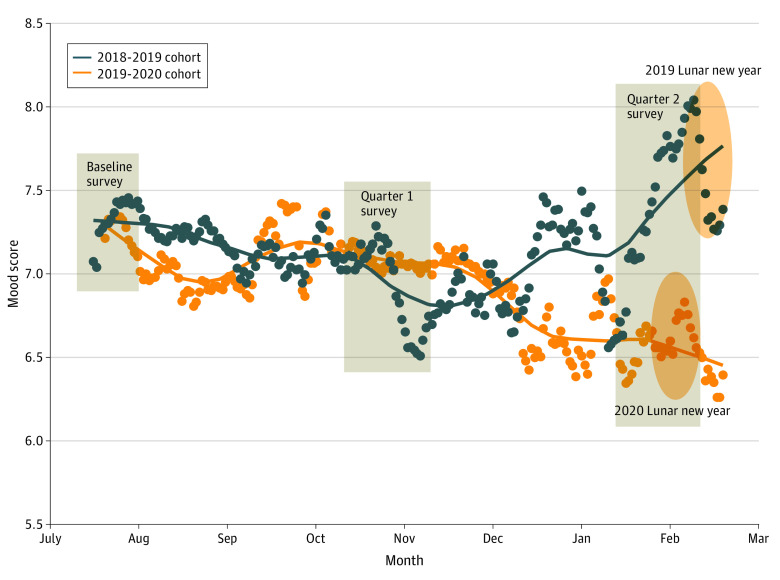
Mood Before and After the Coronavirus Disease 2019 (COVID-19) Outbreak Lines and dots depict daily mood score using a moving weekly mean. The mood score difference between quarter 1 (before the COVID-19 outbreak) and quarter 2 (after the COVID-19 outbreak) was statistically significant for the 2019 to 2020 cohort (β = −0.50; 95% CI, −0.80 to −0.20; *P* = .002). No statistically significant difference was observed for the 2018 to 2019 cohort. The rectangles indicate the time frame for each survey period for the 2019 to 2020 cohort. The ovals indicate the date range for the Lunar New Year holiday for the 2018 to 2019 cohort (February 4, 2019, to February 19, 2019) and 2019 to 2020 cohort (January 24, 2020, to February 8, 2020).

## Discussion

This study found that physicians in China experienced an increase in mental health symptoms and fear of violence and a decline in mood after the COVID-19 outbreak. These findings may reflect training physicians’ added clinical workload with the emergence of COVID-19 and are consistent with past evidence that the additional stressors physicians face during infectious disease outbreaks place them at greater risk for both short-term and long-term mental health problems.^2,3^

A limitation of this study is that our sample consisted of first-year training physicians in China; studies in other physician populations are needed to understand the mental health effects of the COVID-19 pandemic on physicians more broadly. With most new cases now outside China, ensuring that physicians receive appropriate support and access to mental health services is increasingly imperative, for their own well-being, as well as that of their patients and the global community.
